# Effectiveness of a bite-sized web-based intervention to improve healthcare worker wellbeing: A randomized clinical trial of WISER

**DOI:** 10.3389/fpubh.2022.1016407

**Published:** 2022-12-08

**Authors:** J. Bryan Sexton, Kathryn C. Adair, Xin Cui, Daniel S. Tawfik, Jochen Profit

**Affiliations:** ^1^Department of Psychiatry, Duke University School of Medicine, Duke University Health System, Durham, NC, United States; ^2^Duke Center for Healthcare Safety and Quality, Duke University Health System, Durham, NC, United States; ^3^Division of Neonatal and Developmental Medicine, Department of Pediatrics, Stanford University School of Medicine and Lucile Packard Children's Hospital, Palo Alto, CA, United States; ^4^California Perinatal Quality Care Collaborative, Palo Alto, CA, United States; ^5^Division of Pediatric Critical Care Medicine, Department of Pediatrics, Stanford University School of Medicine and Lucile Packard Children's Hospital, Palo Alto, CA, United States

**Keywords:** emotional exhaustion, wellbeing, burnout, wellbeing intervention, bite-sized wellbeing, positive psychology intervention, WISER

## Abstract

**Importance:**

Problems with the wellbeing of healthcare workers (HCWs) are widespread and associated with detrimental consequences for the workforce, organizations, and patients.

**Objective:**

This study tested the effectiveness of the Web-based Implementation for the Science of Enhancing Resilience (WISER) intervention, a positive psychology program, to improve six dimensions of the wellbeing of HCWs.

**Design:**

We conducted a randomized controlled trial of HCWs between 1 April 2018 and 22 July 2019. Cohort 1 received WISER daily for 10 days. Cohort 2 acted as a waitlist control before receiving WISER.

**Setting:**

Web-based intervention for actively employed HCWs across the United States.

**Participants:**

Eligibility criteria included being ≥18 years old and working as a HCW. Each participant was randomized to start the intervention or serve as a waitlist control for 14 days before starting the intervention.

**Interventions:**

Cohorts received links *via* 10 texts exposing them to introductory videos and positive psychology exercises (3 good things, cultivating awe, random acts of kindness, cultivating relationships, and gratitude letters).

**Main outcomes and measures:**

The primary outcome was emotional exhaustion; secondary outcomes included depressive symptoms, work-life integration, happiness, emotional thriving, and emotional recovery. All outcomes were assessed at baseline, 1-week post-intervention (primary endpoint), and 1, 6, and 12-month post-intervention. Outcomes were measured using six validated wellbeing instruments, rescaled to 100-point scales for comparison. Six items assessed participants' WISER experience. The analysis employed mixed-effects models.

**Results:**

In cohorts 1 and 2, 241 and 241 initiated WISER, and 178 (74%) and 186 (77%) completed the 6-month follow-up, respectively. Cohort populations were similar at baseline, mostly female (81; 76%) and nurses (34; 32%) or physicians (22; 23%), with 1–10 years of experience in their current position (54; 52%). Relative to control, WISER significantly improved depressive symptoms [−7.5 (95%CI: −11.0, −4.0), *p* < 0.001], work-life integration [6.5 (95%CI: 4.1, 8.9), *p* < 0.001], happiness [5.7 (95%CI: 3.0, 8.4), *p* < 0.001], emotional thriving [6.4 (95%CI: 2.5, 10.3), *p* = 0.001], and emotional recovery [5.3 (95%CI: 1.7, 8.9), *p* = 0.004], but not emotional exhaustion [−3.7 (95%CI: −8.2, 0.8), *p* = 0.11] at 1 week. Combined cohort results at 1, 6, and 12 months showed that all six wellbeing outcomes were significantly improved relative to baseline (*p* < 0.05 for all). Favorable impressions of WISER were reported by 87% of participants at the 6-month post-assessment.

**Conclusion and relevance:**

WISER improved HCW depressive symptoms, work-life integration, happiness, emotional thriving, and emotional recovery. Improvements in all HCW wellbeing outcomes endured at the 1-, 6-, and 12-month follow-ups. HCW's impressions of WISER were positive.

**Clinical trials number:**

https://clinicaltrials.gov/ct2/show/, identifier: NCT02603133. Web-based Implementation for the Science of Enhancing Resilience Study (WISER).

## Introduction

Safe and reliable healthcare was unambiguously linked to the wellbeing of healthcare workers (HCWs) before there was a global health crisis (1–4). The COVID-19 pandemic has caused historic levels of psychological distress in HCWs in particular (5–8), revealing the additional burdens of social isolation, fear of contracting the disease, economic strain, unpredictable childcare, uncertainty about the future, prolonged bouts of physical and emotional exhaustion, and moral distress. Maintaining a healthy workforce requires not only a sufficient number of HCWs but also maximizing the ability of each one to meet the needs of patients (9).

Before the pandemic, problems with HCW wellbeing were already disturbingly common [e.g., 30–40% of physicians and nurses report burnout (10–12)] and expensive (13), while traditional workplace wellness efforts are costly and often ineffective (14, 15). Longstanding difficulties with work-life integration, challenges with the electronic health record, and a difficult work culture remain largely unaddressed (16–18). Poor HCW wellbeing has been linked to adverse patient events, including increased rates of infections (1, 3) and self-reported errors (1, 2). Furthermore, struggling HCWs are more likely to drop out of the workforce, increasing costly turnover (19–21) and further exacerbating staffing shortages (22).

Although they are not a panacea against the rising tide of HCW burnout, evidence that web-based wellbeing programs are effective is growing (23–26). Unfortunately, feasible interventions to improve wellbeing are uncommon (27). We have developed and refined an engaging, low-burden program [Web-based Implementation for the Science of Enhancing Resilience (WISER)] to enable rapid and enduring improvements in wellbeing (28). This stepwise program uses updated versions of evidence-based interventions drawn from positive psychology that have been effective in improving wellbeing and reducing depression symptoms, which are delivered *via* mobile platforms (24–26, 28).

Wellbeing intervention uptake by busy HCWs requires evidence-based tools that are accessible, easy to use, and engaging. We have adapted WISER considerably in response to these needs (28). In the original WISER trial, we found equivalent efficacy for a 6 vs. 1-month intervention duration. From qualitative feedback, we were encouraged to further reduce the user participation burden. In addition, the original study randomized entire newborn intensive care units. Such clustering (by work setting) may have supported the intervention's efficacy but may limit scalability. Therefore, the objective of this study was to test the efficacy of an abbreviated WISER in 10 texts intervention for improving HCW wellbeing (emotional exhaustion, depressive symptoms, work-life integration, happiness, emotional thriving, and emotional recovery) using an *individual-level randomized design* in which participants were randomized into one of the following two cohorts: the intervention or waitlist control. The efficacy of WISER was tested using the randomized trial 1-week endpoint, but the persistence of effects was tested up to a year later. Given the bite-sized nature of this brief 10-day intervention, evidence supporting the sustainability of improvements to wellbeing is warranted.

Hypothesis 1 (randomized controlled trial, RCT): Efficacy of WISER in 10 texts: The intervention will improve HCW wellbeing compared with waitlist control by the 1-week post-intervention primary endpoint.
Hypothesis 2 (endurance of effects): The benefits of WISER will endure at 1-, 6-, and 12-month post-intervention.

## Materials and methods

### Design

HCWs were randomly assigned to one of the two cohorts in the investigator-initiated, randomized controlled study (RCT) known as WISER in 10 texts. Cohort 1 completed the baseline assessment and received the intervention on 9 July 2018, while cohort 2 acted as a waitlist control and provided an initial (“waitlist control”) assessment on 23 July (14 days later). Cohort 2 completed their baseline assessment and received the intervention. The RCT portion of the study used the 1-week post-assessment results as the primary endpoint to minimize the duration of the waitlist period for HCWs looking to do something about their wellbeing in the near term. The wherewithal necessary to do something about wellbeing of HCWs is increasingly challenging (8). Specifically, this difference-in-differences approach was calculated as follows: (cohort 1: 1-week post-intervention—baseline)—(cohort 2: baseline—waitlist control). A sensitivity analysis using *t*-tests compared 1-week cohort 1 post-intervention with the baseline of cohort 2. The remaining time points were used to test whether benefits endured at 1, 6, and 12 months. The Qualtrics^TM^ platform was used for enrollment, data collection, randomization of individual enrollees to study cohorts, and delivery of text messages with links to WISER activities.

### Participants

The US inpatient and outpatient HCWs were enrolled from 1 April to 9 July 2018 using a link on our website (https://www.hsq.dukehealth.org/), labeled bit.ly/3WISER, or were provided the link during continuing education talks and webinars. Generally, people who seek the content on our website and/or attend our continuing education activities have a background or interest in patient safety, quality improvement, and/or wellbeing. There was also a brief explanation of the WISER intervention during enrollment that provided an overview of the prevalence and severity of wellbeing issues in healthcare, as well as the length and nature of WISER in 10 texts as a method for “pausing and reflecting on what is going well.”

**Figure 1:**
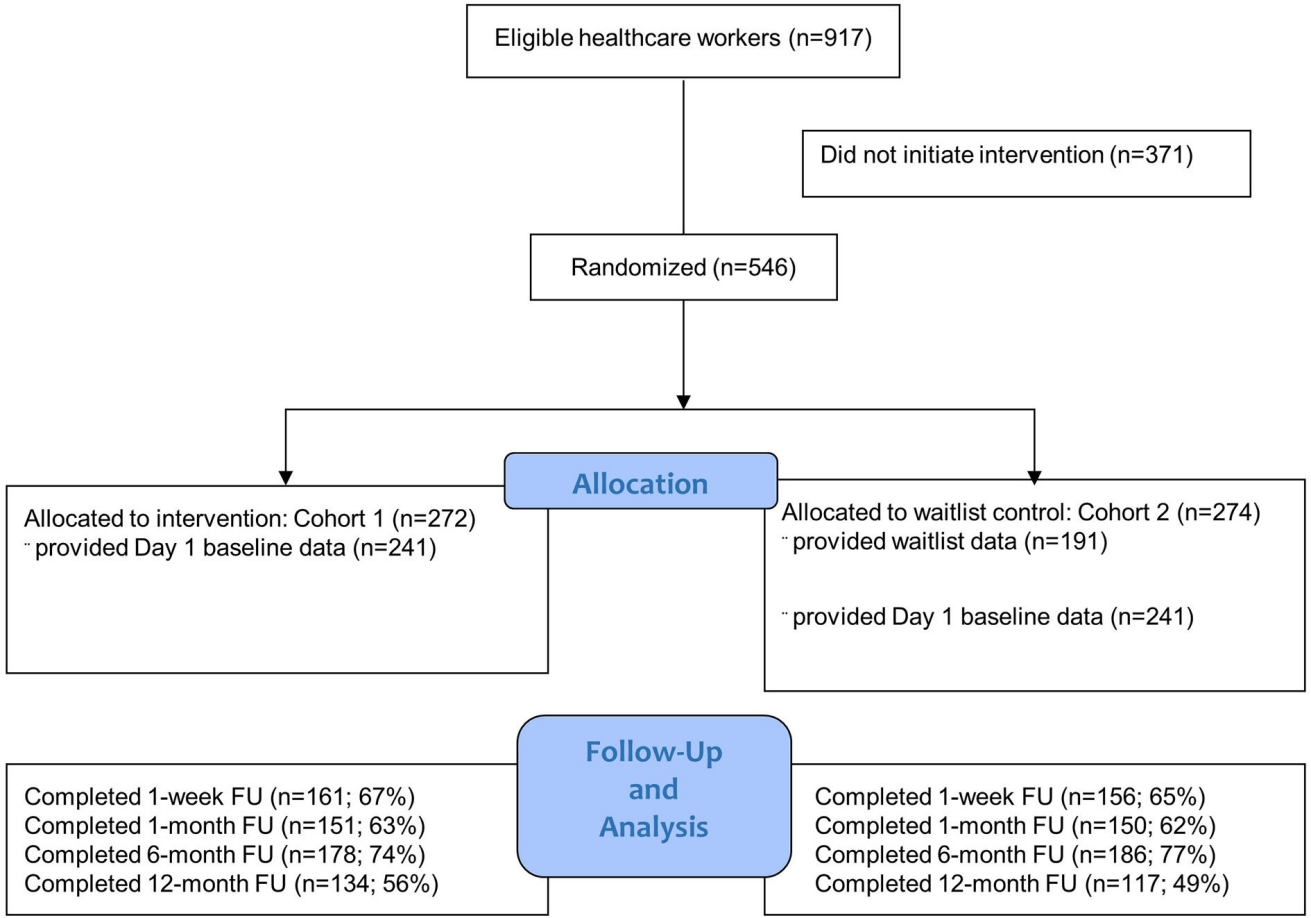
CONSORT diagram.

Participants were informed of the start date and follow-up dates during enrollment. We assessed cohorts at five time points: day 1 (baseline, prior to starting the intervention), and 1, 1, 6, and 12-month post-intervention (Figure 1). Cohort 2 had one additional assessment at the beginning of their waitlist period (titled “waitlist control”) to allow for RCT analyses. Each cohort received the intervention; therefore, blinding was not feasible.

### Intervention

WISER in 10 texts is comprised of five guided wellbeing modules based on adult learning principles, combining educational material with practice-based learning (28). Individual modules have been favorably evaluated as brief, feasible, and practical (24–26). Links to each module of the intervention were delivered *via* text message at 7 pm local time each evening for 10 days. Modules were introduced with an 8–10 min evidence-based educational video and included simple and engaging reflective activities lasting from 2 to 7 min. Every module included a prompt for doing three good things; texts 2–4 and 6–10 included a second “positive reflections” activity to complete. The structure was given as follows:

Text 1: 3 Good Things.
Text 2: 3 Good Things; Cultivate Awe.
Text 3: 3 Good Things; Random Act of Kindness.
Text 4: 3 Good Things; 1 Good Chat/Cultivate Relationships.
Text 5: 3 Good Things; inform the participant that next text they choose the tool they want to use in addition to continuing Three Good Things.
Text 6: 3 Good Things; Choice of Awe/Kindness/ Relationships.
Text 7: 3 Good Things; Choice of Awe/Kindness/ Relationships.
Text 8: 3 Good Things; Choice of Awe/Kindness/ Relationships.
Text 9: 3 Good Things; Choice of Awe/Kindness/ Relationships.
Text 10: 3 Good Things; Cultivate Gratitude.

*Three Good Things* asks participants to reflect on and briefly describe three positive experiences that occurred that day (24, 29). *Cultivate Awe* provided an opportunity to learn about and experience the benefits of awe and wonder through a series of visually and conceptually stunning images followed by an exercise to reflect on one of their own experiences of awe (30). *Random Acts of Kindness* provided an opportunity to learn, practice, and reflect on the power of providing unsolicited kindness to others by documenting acts of kindness that were witnessed, committed, and/or received (31). *Relationship Resilience* promoted an understanding of beneficial relationship patterns, including reflections on recent positive interactions and experiences (32). *Gratitude* provided a structured opportunity to express gratitude toward others through a guided letter-writing exercise (29, 33).

### Measures

The multidimensional nature of wellbeing makes it difficult to summarize in one domain. In addition to the four wellbeing domains (emotional exhaustion, depression, work-life integration, and subjective happiness) used in the original WISER RCT (28), two domains (emotional thriving and emotional recovery) were added. Given the contemporary relevance to HCWs, responsiveness to interventions, and psychometric validity, we chose the primary outcome of emotional exhaustion (EE). EE was assessed by a widely used (8, 16, 17, 24, 25, 34, 35) 5-item derivative of the *emotional exhaustion* scale of the Maslach Burnout Inventory (36), shown to have excellent psychometric properties (24–26, 34, 35, 37, 38), external validity (16, 17, 34), and responsiveness to interventions (18, 24–26, 37). Details of each wellbeing domain are in the Supplementary material.

### Randomization

Participants enrolled using Qualtrics, which consecutively randomized participants into two cohorts. Participants received details of the intervention and their start date in an email for their records.

### Statistical analyses

For comparability across the six wellbeing domains, we rescaled outcome measures to 100-point scales. Hypotheses tested included the efficacy of WISER in cohort 1 (intervention) vs. cohort 2 (waitlist control) and combined cohort changes from baseline to 1-week, 1-month, 6-month, and 12-month post-assessments. The randomized trial and endurance of effects portions of the study were evaluated using generalized linear mixed-effects modeling that included fixed effects for time and random effects for participants to account for within-participant correlation (39). To facilitate statistical power and interpretation of the long-term follow-up results, we combined the two cohorts and used percent concerning thresholds. This technique is commonly used in safety culture and wellbeing research when looking across a set of metrics (some positively and some negatively balanced) such that a “low percent concerning,” or a reduction in percent concerning was easier to interpret (16, 17, 34, 40).

Mixed-model hypothesis tests were conducted in SAS 9.4 using PROC GLIMMIX. A *p*-value of <0.05 was considered statistically significant. We considered EE improvement of at least 10% from baseline to be meaningful based on previous studies (24, 26, 28, 41). This translated to a decrease in EE from 50/100 to 45/100, a 5-point decline, or an effect size of 0.25, assuming a standard deviation of 20, needing 253 participants in each arm of the intervention to have 80% power to detect this effect size.

## Results

Enrollment and participation in the trial are shown in Figure 1 (the CONSORT diagram). In cohort 1, 241 respondents initiated the intervention, with 161 (67%) in the follow-up at 1 week, 151 (63%) at 1 month, 178 (74%) at 6 months, and 134 (56%) at 12 months. In cohort 2, 241 respondents initiated the intervention, with 156 (65%) in the follow-up at 1 week, 150 (62%) at 1 month, 186 (77%) at 6 months, and 117 (49%) at 12 months. Table 1 displays the characteristics of the study population by cohort prior to the intervention. Cohorts 1 and 2 had similar demographics at baseline. No adverse events were reported. The six dimensions of HCW wellbeing exhibited good psychometric reliability (Cronbach's α): emotional exhaustion (α = 0.84), depression (α = 0.83), happiness (α = 0.86), work-life integration (α = 0.80), emotional thriving (α = 0.81), and emotional recovery (α = 0.82).

**Table 1:** Characteristics of the study population before intervention.

	**Cohort 1**				**Cohort 2**					
	**Enrollment**		**Baseline** ^*^		**Enrollment**		**Waitlist** ^**^		**Baseline** ^*^	
	***n***	**%**	***n***	**%**	***n***	**%**	***n***	**%**	***n***	**%**
Total	272	100.0	241	100.0	274	100.0	191	100.0	241	100.0
**Sex**										
Male	25	9.2	23	9.5	39	14.2	26	13.6	35	14.5
Female	213	78.3	196	81.3	203	74.1	152	79.6	184	76.3
**Race/ethnicity**										
Hispanic of any race	11	4.0	11	4.6	16	5.8	11	5.8	15	6.2
White	233	85.7	208	86.3	228	83.2	157	82.2	199	82.6
African American	^†^		^†^		6	2.2	6	3.1	6	2.5
Asian	20	7.4	16	6.6	24	8.8	17	8.9	21	8.7
Others	^†^		^†^		0	0.0	0	0.0	0	0.0
**Typical shift length**										
8 h	73	26.8	65	27.0	65	23.7	47	24.6	60	24.9
10 or 12 h	110	40.4	106	44.0	137	50.0	102	53.4	120	49.8
24 h	7	2.6	6	2.5	7	2.6	^†^		7	2.9
Others	24	8.8	20	8.3	15	5.5	13	6.8	15	6.2
**Healthcare worker role**										
Physician^a^	62	22.8	52	21.6	64	23.4	38	19.9	55	22.8
Nurse^b^	92	33.8	83	34.4	92	33.6	63	33.0	77	32.0
APP^c^	13	4.8	13	5.4	17	6.2	11	5.8	16	6.6
Others^d^	105	38.6	93	38.6	100	36.5	78	40.8	92	38.2
**Work experience in current position**										
< 1 year	27	9.9	23	9.5	31	11.3	22	11.5	28	11.6
1–10 years	92	33.8	130	53.9	137	50.0	101	52.9	126	52.3
≥ 11 years	13	4.8	71	29.5	76	27.7	55	28.8	67	27.8
**Specialty**										
Surgical^e^	15	5.5	13	5.4	8	2.9	^†^		8	3.3
Critical Care/ER^f^	51	18.8	50	20.7	74	27.0	54	28.3	62	25.7
Pediatrics (non-NICU)	19	7.0	17	7.1	23	8.4	14	7.3	19	7.9
Others^g^	146	53.7	132	54.8	126	46.0	98	51.3	119	49.4
**Typical work in**										
Adult	77	28.3	75	31.1	54	19.7	36	18.8	48	19.9
Pediatrics	99	36.4	92	38.2	115	42.0	89	46.6	101	41.9
Both	34	12.5	29	12.0	50	18.2	32	16.8	47	19.5
Not applicable	30	11.0	24	10.0	21	7.7	19	9.9	21	8.7
Inpatient	126	46.3	117	48.5	136	49.6	98	51.3	124	51.5
Outpatient	82	30.1	77	32.0	75	27.4	56	29.3	67	27.8
Not applicable	30	11.0	24	10.0	25	9.1	19	9.9	24	10.0
**Outcome**	**Cohort 1**				**Cohort 2**					
	**Enrollment**		**Baseline** ^*^		**Enrollment**		**Waitlist** ^**^		**Baseline** ^*^	
**100-point scale [*n*, mean (SD)]**										
Emotional exhaustion	272	57.1 (26.3)	241	54.8 (25.8)	273	59.2 (25.8)	191	58.9 (26.3)	222	56.9 (25.3)
Depressive symptoms	257	31.8 (18.5)	209	33.1 (19.8)	260	32.3 (18.2)	182	32.7 (18.2)	200	30.4 (18.8)
Work-life integration	272	67.9 (15.3)	222	73.4 (13.1)	272	67.0 (15.6)	191	73.3 (12.6)	210	73.7 (13.3)
Happiness	272	66.3 (20.2)	222	62.0 (22.7)	274	62.0 (19.5)	191	61.4 (20.1)	209	60.9 (20.1)
Emotional recovery	272	67.1 (22.0)	228	65.8 (22.6)	273	66.0 (21.0)	191	67.3 (22.0)	222	67.3 (20.1)
Emotional thriving	272	67.0 (24.0)	228	67.2 (23.7)	273	66.8 (22.5)	191	67.2 (22.8)	222	66.2 (23.3)
**Percent concerning rate** ^h^										
Emotional exhaustion	64.3		63.1		68.5		67.5		67.1	
Depressive symptoms	35.0		43.1		37.3		41.2		39.0	
Work-life integration	57.4		46.4		65.1		45.6		42.9	
Happiness	58.5		66.7		67.2		69.1		70.3	
Emotional recovery	50.4		57.5		52.4		49.7		51.8	
Emotional thriving	51.1		51.8		53.5		52.8		51.8	

a Physician includes attending, staff, fellow, and resident physician.

b Nurse includes registered nurse, nurse manager, and charge nurse.

c Advance practice provider (APP) includes physician assistant and nurse practitioner.

d Other roles include therapist (e.g., respiratory, physical, occupational, and speech therapist), administrative support (e.g., clerk, secretary, and receptionist), clinical support (e.g., CMA and nurses aid), pharmacist, clinical social worker, manager, dietician/nutritionist, student, and others.

e Surgical specialties include anesthesiology, obstetrics and gynecology, and surgery.

f High-intensity medical care specialties include emergency medicine, critical care medicine, and NICU.

g Other specialties include family practice, internal medicine, neurology, physical medicine & rehabilitation, preventive medicine, psychiatry, radiology, and others.

h Percent concerning rates were calculated using previously published thresholds.

* Baseline defined as the first day of intervention.

** Cohort 2 served as waitlist control, while cohort 1 started the intervention.

† Categories with ≤ 5 individuals are not reported in order to protect subject privacy. Data may not add up to 100% due to missing data.

*RCT Efficacy: The intervention will improve HCW's emotional exhaustion, depression, work-life integration, happiness, emotional thriving, and emotional recovery compared with* the waitlist control by the 1-week post-intervention primary endpoint (Hypothesis 1). On a 100-point scale, compared with cohort 2 (waitlist control), the WISER intervention in cohort 1 reduced depression (−7.5, 95% CI −11.0–−4.0, *p* <0 .001) and improved work-life integration (6.5, 95% CI 4.1–8.9, *p* < 0.001), happiness (5.7, 95% CI 3.0–8.4, *p* < 0.001), emotional thriving (6.4, 95% CI 2.5–10.3, *p* = 0.004), and emotional recovery (5.3, 95% CI 1.7–8.9, *p* < 0.001), but emotional exhaustion did not reach significance (−3.7, 95% CI −8.2–0.8, *p* = 0.11; see Table 2). The sensitivity analysis using *t*-tests compared 1-week cohort 1 post-intervention with the baseline of cohort 2 showing significant improvements in all wellbeing outcomes (*p* ≤ 0.005) except for emotional thriving and emotional recovery (*p* = 0.13; see Table 2).

**Table 2:** Efficacy of WISER intervention at 1 week in mixed-effects model^a^ and sensitivity analyses^b^.

	**Mixed effects model**		**Sensitivity (*t*-test)**	
	**Estimate (95% CI)**	***P*-value**	**Estimate (95% CI)**	***P*-value**
Emotional exhaustion	−3.7 (−8.2, 0.8)	0.11	−8.8 (−14.5, −3.1)	0.003
Depressive symptoms	−7.5 (−11.0, −4.0)	<0.001	−10.7 (−14.3, −7.1)	<0.001
Work-life integration	6.5 (4.1, 8.9)	<0.001	8.8 (6.2, 11.3)	<0.001
Happiness	5.7 (3.0, 8.4)	<0.001	6.1 (1.9, 10.4)	0.005
Emotional recovery	5.3 (1.7, 8.9)	0.004	3.7 (−1.1, 8.5)	0.130
Emotional thriving	6.4 (2.5, 10.3)	0.001	3.8 (−1.1, 8.7)	0.129

a Efficacy of WISER: The intervention improves healthcare workers' emotional exhaustion, depressive symptoms, work-life integration, happiness, emotional recovery, and emotional thriving in cohort 1 compared with waitlist control in cohort 2. (C1: 1 week – baseline) – (C2: baseline – waitlist).

b The sensitivity analysis using t-tests compared 1-week cohort 1 post-intervention with the baseline of cohort 2 (both assessments occurred on the same day).

*Endurance of effects: The effects of WISER will endure at 1-, 6-, and 12-month post-intervention (Hypothesis 2)*.

**Figure 2:**
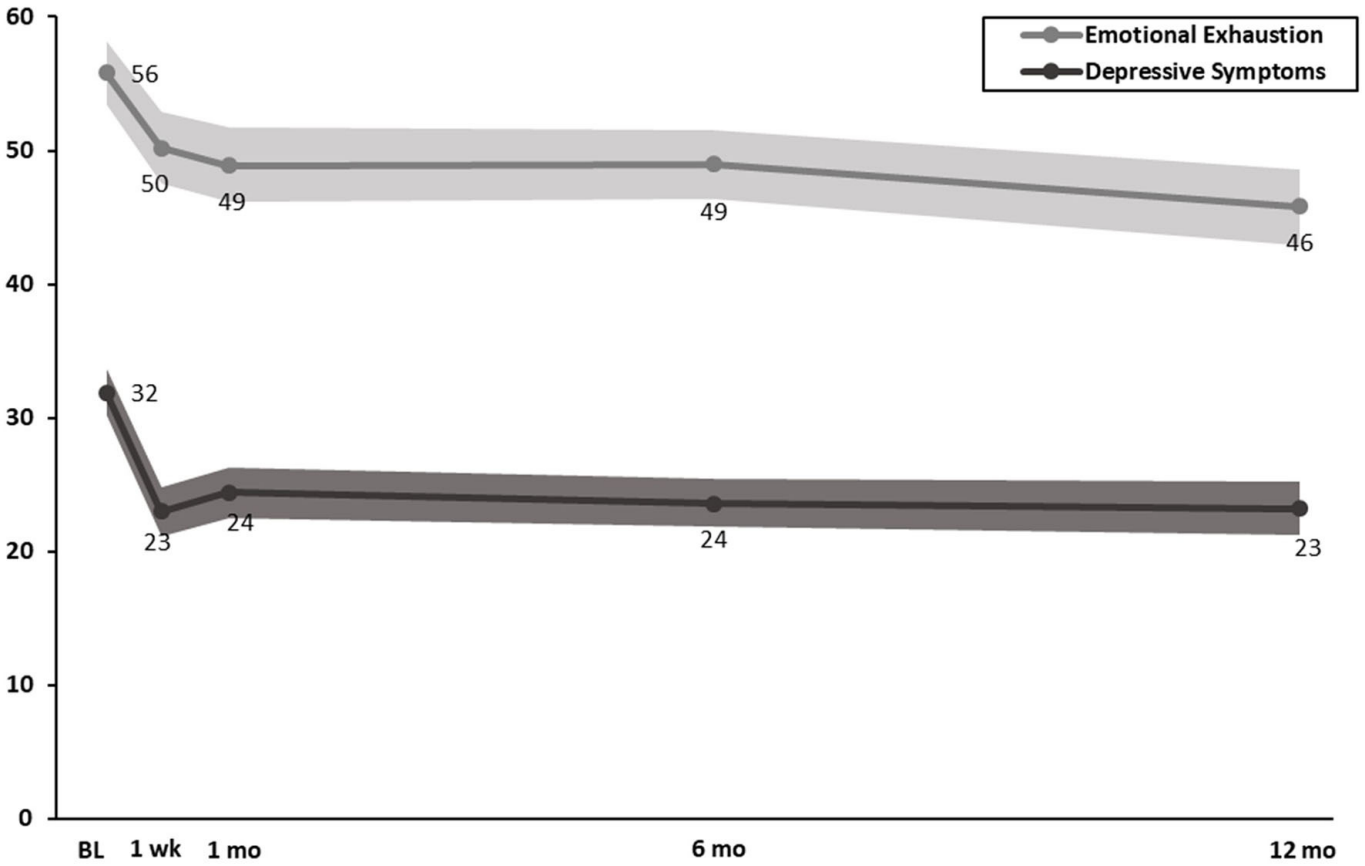
Effect of WISER for emotional exhaustion and depressive symptoms at 1-week, 1-, 6-, and 12-month post-intervention (100-point scale). Dots in the middle line: point estimates from mixed-effects models for each time point; shaded areas: 95% confidence intervals from mixed-effects models. BL, baseline; 1 wk, 1-week post-intervention; 1 mo, 1-month post-intervention; 6 mo, 6-month post-intervention; 12 mo, 12-month post-intervention.

**Figure 3:**
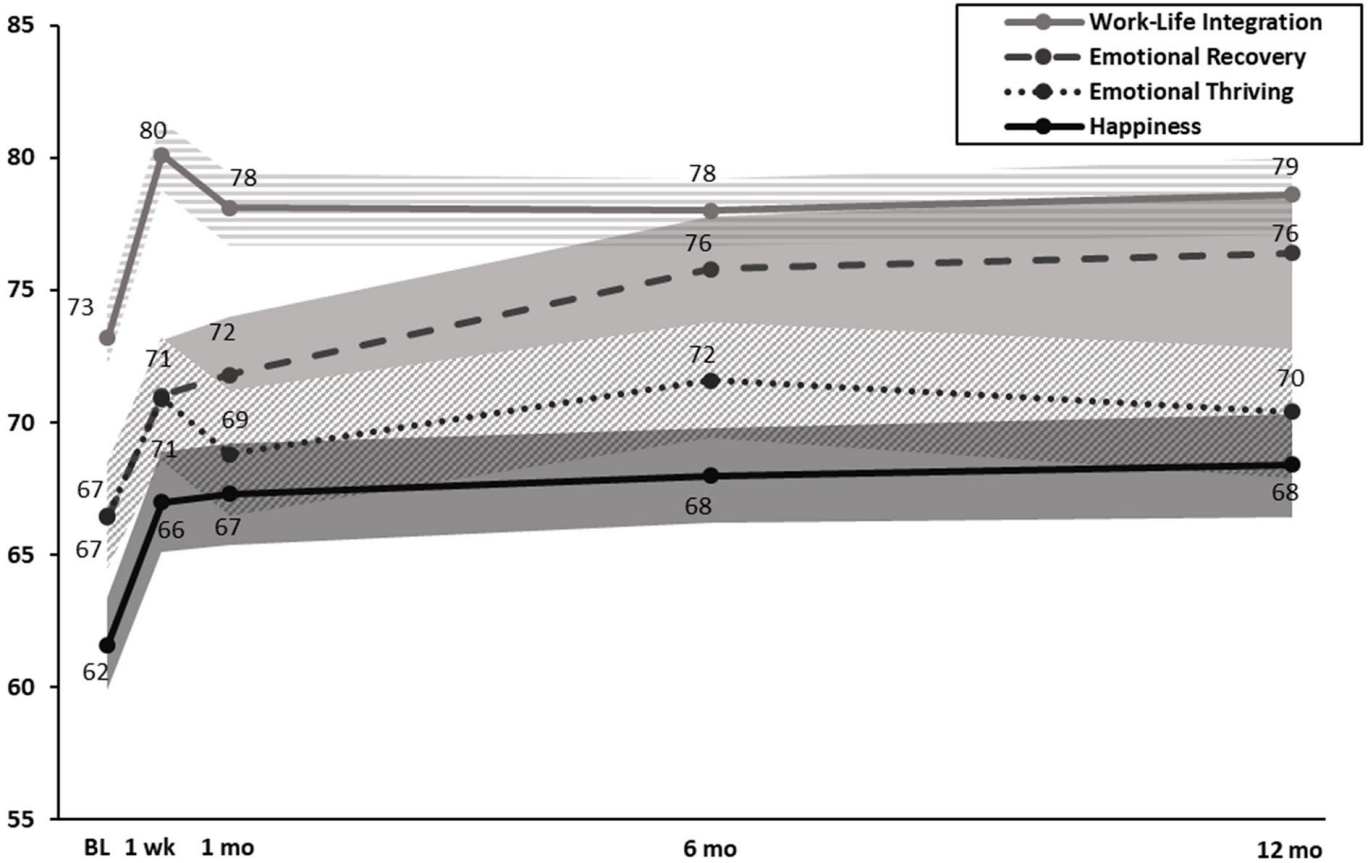
Effect of WISER for work-life integration, emotional recovery, emotional thriving, and happiness at 1-week, 1-, 6-, and 12-month post-intervention (100-point scale). Note: Dots in the middle line: point estimates from mixed-effects models for each time point; shaded areas: 95% confidence intervals from mixed-effects models. BL, baseline; 1 wk, 1-week post-intervention; 1 mo, 1-month post-intervention; 6 mo, 6-month post-intervention; 12 mo, 12-month post-intervention.

In combined cohorts on a 100-point scale, at 1-, 6-, and 12-month post-intervention, WISER was associated with reduced emotional exhaustion (*p* < 0.001), reduced depressive symptoms (*p* < 0.001), improved work-life integration (*p* < 0.001), improved happiness (*p* < 0.001), improved emotional recovery (*p* < 0.001), and improved emotional thriving (*p* < 0.001 to 0.03), relative to baseline (Table 3; Figures 2, 3). In combined cohorts using % concerning, WISER was similarly associated with improvements in all outcomes at all time points (Table 4).

**Table 3:** Effectiveness of WISER at 1-week, 1-, 6-, and 12-month post-intervention (100-point scale), combined cohorts.

**1-week**		**1-month**		**6-month**		**12-month**	
**Estimate (95%CI)**	***P*-value**	**Estimate (95%CI)**	***P*-value**	**Estimate (95%CI)**	***P*-value**	**Estimate (95%CI)**	***P*-value**
**Emotional exhaustion**							
−5.6 (−8.1, −3.2)	<0.001	−6.9 (−9.4, −4.4)	<0.001	−6.9 (−9.2, −4.6)	<0.001	−10.0 (−12.6, −7.4)	<0.001
**Depressive Symptoms**							
−8.9 (−10.8, −7.1)	<0.001	−7.5 (−9.3, −5.6)	<0.001	−8.3 (−10.0, −6.5)	<0.001	−8.7 (−10.7, −6.7)	<0.001
**Work-life integration**							
6.8 (5.6, 8.1)	<0.001	4.8 (3.5, 6.1)	<0.001	4.7 (3.5, 6.0)	<0.001	5.3 (3.9, 6.7)	<0.001
**Happiness**							
5.4 (3.9, 6.8)	<0.001	5.7 (4.3, 7.1)	<0.001	6.4 (5.0, 7.8)	<0.001	6.7 (5.2, 8.3)	<0.001
**Emotional recovery**							
4.6 (2.7, 6.5)	<0.001	5.5 (3.5, 7.4)	<0.001	9.4 (7.6, 11.2)	<0.001	10.1 (8.0, 12.1)	<0.001
**Emotional thriving**							
4.5 (2.4, 6.5)	<0.001	2.3 (0.2, 4.5)	0.03	5.1 (3.1, 7.1)	<0.001	3.9 (1.6, 6.1)	0.001

**Table 4:** Effectiveness of WISER at 1-week, 1-, 6-, and 12-month post-intervention (% concerning), combined cohorts.

**1-wk**		**1-mo**		**6-mo**		**12-mo**	
**Estimate (95%CI)**	***P*-value**	**Estimate (95%CI)**	***P*-value**	**Estimate (95%CI)**	***P*-value**	**Estimate (95%CI)**	***P*-value**
**Emotional exhaustion**							
−14.3 (−22.2, −6.4)	0.002	−22.6 (−31.4, −13.5)	0.002	−17.6 (−25.6, −9.1)	0.002	−24.1 (−34.4, −14.3)	0.002
**Depressive symptoms**							
−35.6 (−44.5, −28.4)	0.002	−27.4 (−36.5, −19.0)	0.002	−33.1 (−40.9, −24.2)	0.002	−32.6 (−41.2, −23.1)	0.002
**Work-life integration**							
−33.9 (−41.7, −25.8)	0.002	−23.9 (−32.9, −15.1)	0.002	−24.6 (−32.7, −16.2)	0.002	−27.0 (−36.6, −18.0)	0.002
**Happiness**							
−16.5 (−24.7, −9.2)	0.002	−14.0 (−22.8, −5.9)	0.002	−18.2 (−25.9, −11.2)	0.002	−22.6 (−31.5, −13.9)	0.002
**Emotional recovery**							
−24.0 (−32.9, −15.4)	0.002	−25.2 (−35.4, −15.6)	0.002	−35.3 (−45.4, −27.1)	0.002	−38.0 (−47.8, −29.2)	0.002
**Emotional thriving**							
−13.8 (−21.6, −4.7)	0.004	−8.6 (−17.2, −0.2)	0.02	−15.4 (−24.9, −6.9)	0.002	−12.8 (−23.3, −2.3)	0.014

Neither RCT efficacy nor endurance of effects results changed meaningfully after adjusting for covariates.

Participant evaluation of WISER at the 6-month post-intervention was positive, with 87% reporting overall favorable impressions (see Supplementary material for Participant Evaluation of WISER).

## Discussion

In this RCT, WISER in 10 texts demonstrated robust evidence of efficacy across 5 of 6 wellbeing outcome measures by 1 week and was associated with enduring improvements for all 6 wellbeing outcomes at the 1-, 6-, and 12-month post-assessment of emotional exhaustion, depressive symptoms, work-life integration, happiness, emotional thriving, and emotional recovery. WISER demonstrated a significant and enduring effect among neonatal intensive care unit HCWs in the original RCT (28), and here we find similar results despite a significantly compressed intervention period from 6 months to only 10 days. The magnitude of the program's wellbeing improvements is favorable relative to a meta-analysis of interventions to improve HCW wellbeing (27).

Implementation of WISER demonstrated efficacy for 5 of the 6 outcomes at the 1-week post-intervention (emotional exhaustion did not reach statistical significance), and it demonstrated enduring effects for all outcomes at all subsequent follow-up time points. Emotional exhaustion was significantly lower by the 1-month follow-up and that reduction continued at 6 and 12 months. In retrospect, our efforts to shorten WISER, including the follow-up period of 1-week post-intervention, may have been too early to detect a significant change in emotional exhaustion. Previous studies using the 1-month follow-up timeframe have shown significant reductions in HCW emotional exhaustion (24–26, 28), and it may take several weeks for the increases in access to positive emotions attained through WISER to accrue. Although the mixed effects model RCT analysis of emotional exhaustion at 1 week did not reach significance, the added statistical power to detect improvements in combined cohort analyses (Tables 3, 4) demonstrated *significant improvements for all wellbeing outcomes across all time periods* (including the 1-week post-intervention). The first RCT of WISER used a 1-month post-intervention and found that WISER reduced emotional exhaustion by 1 month, and the effect continued at the 6-month follow-up. Future studies should consider extending the post-intervention beyond 1 week when EE is used.

Wellbeing interventions based on positive psychology (42) research may help alleviate some of the growing problems with HCWs' wellbeing, which is especially salient during the unprecedented and prolonged demands of the COVID-19 pandemic (5, 8, 43). The durability and increase of effect sizes in wellbeing outcomes over time suggest that our results are clinically meaningful and compare favorably with longer and more resource-dependent interventions intended to improve wellbeing and mental health, such as individualized coaching or meditation (41, 44–46). The HCW WISER user experience was positive, with 87% reporting overall favorable impressions. Consistent with the first RCT (28) of WISER showing improvements at 1- and 6-month post-intervention, the current RCT showed that a simple, low-resource intervention can cause robust improvements in individual HCW wellbeing that are enduring *for at least a year*. As of this writing, WISER in 10 texts is still available as a research study to all US HCWs at bit.ly/3WISER.

Notwithstanding the efficacy of WISER, we wish to avoid a false dichotomy on how to address HCW wellbeing. It is tempting to polarize the topic of individual vs. institutional resources for HCW wellbeing and to advocate for institutional reforms almost exclusively (47). Understandably frustrated HCWs who have experienced repeated upheavals from broken systems are correct to insist on improvements. However, this mindset only focuses on part of the solution because we *also* need individual-level solutions for the plurality of HCWs (5) who are struggling with wellbeing *right now* (48). Fixing the system takes time, and obvious targets for sources of burnout such as the electronic health record account for less than one-tenth of the burnout variance in comparison to work culture (18). A more practical, responsive, and nuanced approach would be to “empower HCWs to fix the system *and* give them some useful wellbeing resources to choose from because they are profoundly stressed here and now.” This dual approach would also be responsive to the significant differences in wellbeing by HCW role, gender, years of experience, specialty, and individual proclivity (8).

Most participants (87%) reported that WISER helped them to recognize more opportunities for positive emotions, potentially elucidating the psychological pathways that underlie WISER's effectiveness. People who struggle with wellbeing experience decreased ability to notice and pay attention to positive stimuli (49). Rigorous psychological research has consistently shown that experiencing positive emotion is central to building consequential personal resources like wellbeing (50), as well as helping to find meaning after adversity (51), and accelerating recovery after emotional upheavals (52). Positive emotions like hope, gratitude, and serenity may recharge depleted batteries (53), and experiencing positive emotions has both psychological and physiological benefits (54). Despite the well-documented descriptions of burnout in healthcare, few interventions have been tested in randomized trials, and fewer still have used multiple theory-driven interventions. The WISER intervention packages tools that promote noticing and savoring positive emotions, require only a mobile phone, and is scalable and free, and its 10-day version is quite feasible.

## Limitations

This study should be viewed in light of its design. In line with other wellbeing behavioral intervention studies (55–60), we experienced non-initiation and attrition in both study cohorts, which may introduce selection bias. The number of participants who initiated the intervention by providing baseline data (*n* = 482) was considerably smaller than the number who expressed interest (*n* = 917) in this study by clicking the study description link, meaning 53% initiated WISER. This challenge of initiation rates among busy HCWs was exemplified in a recent innovative RCT of professional coaching (41) for physicians that showed similar efficacy to our study, yet with only 88 of 764 (11.5%) eligible physicians choosing to participate. This study compares favorably to this and other interventions, including dieting, smoking cessation, and other web-based wellbeing interventions (55, 56, 61), which tend to have low rates of initiation (~20%) even when financial incentives are provided (55). Similarly, the Centers for Disease Control and Prevention recognized in-person lifestyle change programs such as the National Diabetes Prevention Program average over 60% attrition by week 44 (62), and web-based weight-loss programs report 65–70% of their initiators are no longer using the web-based program by the 52-week mark (63). Emotional exhaustion itself may contribute to a lack of initiation energy (8) and may explain the lack of effectiveness found in workplace wellbeing programs (14). It is unknown if participants who were lost to follow-up experienced similar improvements to those who completed the study, suggesting that our results should be interpreted with caution but those who completed the intervention derived significant benefits. Statistical power was good for the combined cohorts but slightly underpowered for individual cohort analyses (this could explain why emotional exhaustion did not reach significance at 1-week by cohort but did in the combined cohort analyses). This lack of statistical power to compare individual cohorts across follow-ups is a significant limitation, and future studies should recruit to the point of overpowered samples given the growing problems of loss to follow-up in HCW wellbeing studies. Using an enrollment process with a fixed starting date provided predictability for participants, but future studies may consider rolling enrollments and start dates to allow for adequately powered analyses to be used. Finally, our participants were mostly white females, which reflects the workforce demographics in many large academic centers. We know that female HCWs report significantly worse wellbeing than their male counterparts (64), and that female, racial, and ethnic minority HCWs experience more mistreatment and discrimination by patients, families, and visitors in ways that deteriorate wellbeing (65). It is uncertain whether our findings are generalizable to settings with more diverse workforces, and the extent to which particular HCW demographic groups benefit from wellbeing interventions is an area ripe for future research.

## Conclusion

WISER in 10 texts significantly improved the wellbeing of HCWs relative to a waitlist control, and improvements were sustained over 1 year for emotional exhaustion, depressive symptoms, work-life integration, happiness, emotional thriving, and emotional recovery. Participants reported enjoyment of the intervention, with four out of five evaluating WISER favorably. Although initiating the intervention among busy HCWs can be a challenge, WISER provides an ongoing low-intensity positive psychology wellbeing resource as HCWs continue to respond to the COVID-19 pandemic.

## Data availability statement

The authors will share raw data from the study upon reasonable request to the corresponding author.

## Ethics statement

The studies involving human participants were reviewed and approved by Duke University Institutional Review Board. The patients/participants provided their written informed consent to participate in this study.

## Author contributions

JS created WISER, conceptualized and designed the study, assisted in data analysis and interpretation, critically reviewed the manuscript, and approved the final manuscript as submitted. KA consulted on tool development and study design, assisted in the analysis and interpretation of the data, critically reviewed the manuscript, and approved the final manuscript as submitted. XC coordinated the data management and carried out the initial data analyses, critically reviewed the manuscript, and approved the final manuscript as submitted. DT assisted in the interpretation of the data, critically reviewed the manuscript, and approved the final manuscript as submitted. JP conceptualized and designed the study, assisted in data analysis and interpretation, critically reviewed the manuscript, and approved the final manuscript as submitted. All authors agreed to be accountable for all aspects of the work in ensuring that questions related to the accuracy or integrity of any part of the work are appropriately investigated and resolved.
