# Supplementary material for: Effectiveness of a bite-sized web-based intervention to improve healthcare worker wellbeing: A randomized clinical trial of WISER
Source: Front Public Health. 2022 Dec 8;10:1016407. doi: 10.3389/fpubh.2022.1016407 (PMC9773843; doi:10.3389/fpubh.2022.1016407)
Supplement: Supplementary file 1 [file Data_Sheet_1.PDF]

Supplementary Material

I. Measures Details
II. Participant Evaluation of WISER
III. Supplementary Table 1. Effectiveness of WISER at 1-week, 1-, 6-, 12-month post intervention (% concerning), combined cohorts

I. Measures Details

Primary Outcome

Emotional Exhaustion. According to a psychometric meta-analysis, of the three sub-scales of burnout (emotional exhaustion, depersonalization, and personal accomplishment), EE consistently produces the largest and most consistent coefficient alpha estimates (1). Only EE has shown adequate reliability for individual-level measurement (2), and furthermore, it discriminates between burned out and non-burned out outpatients suffering from work-related neurasthenia (3,4), (according to the International Statistical Classification of Diseases and Related Health Problems, 10th revision (ICD-10) criteria and Diagnostic and Statistical Manual of Mental Disorders, 5th edition-(DSM-IV). For ease of interpretability, we defined a “percent concerning” measure to highlight the proportion of respondents in each cohort reporting undesirable results. We used the established threshold of 50 or higher (5–9), which reflects “not disagreeing,” on average, to emotional exhaustion items. Example item: Events at work affect my life in an emotionally unhealthy way; from 1 (disagree strongly) to 5 (agree strongly).

Secondary Outcomes

The remaining five dimensions of well-being were depressive symptoms, work-life integration, subjective well-being, emotional thriving and emotional recovery.

Depressive symptoms were assessed via the Center for Epidemiological Studies Depression Scale-10-item version (CES-D10), a psychometrically sound (5,6,10) tool for screening respondents for clinical depression (11). This widely used and reliable metric is responsive to well-being interventions, including the original series of RCTs to validate several of the positive psychology interventions (12), and during our subsequent adaptations of these interventions for use by HCWs (5,6,10,13). We have also used the CES-D10 with HCWs to demonstrate links to disruptive behaviors, emotional exhaustion, and work-life integration (9,14). CES-D10 items are prefaced with, "during the past week, how often did this occur" and include items such as "I could not 'get going'" and "my sleep was restless"; from 0=rarely or none to 3=all of the time. Individual responses are summed together to achieve a 0-30 point scale.

Work-life integration was evaluated using the work-life climate scale, which has been used with HCWs to demonstrate good psychometrics (7,15,16), and responsiveness to interventions (5,6,10). Items elicit behavioral work-life infractions by asking: "During the past week, how often did this occur?", followed by phrases such as: arrived home late from work or slept less than five hours in a night. Response options were 1= Rarely or none of the time (less than 1 day); 2=Some or a little of the time (1-2 days); 3=Occasionally or a moderate amount of time (3-4 days); 4=All of the time (5-7 days); and Not Applicable. Work-life climate scale scores were computed by taking the mean of the eight items (maximum score of 4). We first reversed these scores such that higher scores are representative of better work-life integration.

Subjective Happiness was evaluated with the subjective happiness scale, a validated, psychometrically sound, and internationally used scale of global happiness (17,18), also responsive to interventions (5,6,10).

Emotional thriving assesses the level of flourishing of a respondent, capturing, in essence, the opposite of emotional exhaustion, using positively valenced items.

Emotional recovery captures extent to which one is ready to “bounce back,” from adversity or emotional upheavals. Emotional recovery and emotional thriving only share about 15% of their variance at the work setting level, and 10% at the individual level, so being good at recovery does not ensure thriving, and vice versa. Both scales have Cronbach’s alphas of .89, and are responsive to interventions (10). The survey also captured respondent characteristics including gender, race/ethnicity, shift type, job position, and years in specialty. Job positions included attending physician, fellow (trainee) physician, nurse practitioner, registered nurse, respiratory care practitioner, and other. At the 6-month post assessment, six items (using a 5 point Likert scale from disagree strongly to agree strongly) evaluated participant experience with WISER. The percent of participants that agreed (slightly or strongly) to the evaluation questions is provided.

II. Participant evaluation of WISER

364 participants reported on satisfaction, meaningfulness and overall impressions of WISER at the 6-month post assessment. Of these, 87% reported “My overall impression of WISER was favorable.” 87% reported “WISER helped me to recognize more opportunities for positive emotions”; and 80% reported “The evidence based nature of WISER was important to me.” 79% reported “I would be interested in other programs like WISER.” 77% reported “WISER helped me to experience more positive emotions.” 76% reported “WISER demonstrated activities that were meaningful to me personally.”

EAppendix Table 1. Effectiveness of WISER at 1-week, 1-, 6-, 12-month post intervention (% concerning), combined cohorts

1-wk		1-mo		6-mo		12-mo	
Estimate (95%CI)	P-value	Estimate (95%CI)	P-value	Estimate (95%CI)	P-value	Estimate (95%CI)	P-value
			**Emotional Exhaustion**				
-14.3 (-22.2, -6.4)	.002	-22.6 (-31.4, -13.5)	.002	-17.6 (-25.6, -9.1)	.002	-24.1 (-34.4, -14.3)	.002
			**Depressive Symptoms**				
-35.6 (-44.5, -28.4)	.002	-27.4 (-36.5, -19.0)	.002	-33.1 (-40.9, -24.2)	.002	-32.6 (-41.2, -23.1)	.002
			**Work-Life Integration**				
-33.9 (-41.7, -25.8)	.002	-23.9 (-32.9, -15.1)	.002	-24.6 (-32.7, -16.2)	.002	-27.0 (-36.6, -18.0)	.002
			**Happiness**				
-16.5 (-24.7, -9.2)	.002	-14.0 (-22.8, -5.9)	.002	-18.2 (-25.9, -11.2)	.002	-22.6 (-31.5, -13.9)	.002
			**Emotional Recovery**				
-24.0 (-32.9, -15.4)	.002	-25.2 (-35.4, -15.6)	.002	-35.3 (-45.4, -27.1)	.002	-38.0 (-47.8, -29.2)	.002
			**Emotional Thriving**				
-13.8 (-21.6, -4.7)	.004	-8.6 (-17.2, -0.2)	.02	-15.4 (-24.9, -6.9)	.002	-12.8 (-23.3, -2.3)	.014

References

1. Wheeler DL, Vassar M, Worley JA, Barnes LLB. A Reliability Generalization Meta-Analysis of Coefficient Alpha for the Maslach Burnout Inventory. Educational and Psychological Measurement. 2011 Feb;71(1):231–44.

2. Brady KJS, Ni P, Sheldrick RC, Trockel MT, Shanafelt TD, Rowe SG, et al. Describing the emotional exhaustion, depersonalization, and low personal accomplishment symptoms associated with Maslach Burnout Inventory subscale scores in US physicians: an item response theory analysis. Journal of Patient-Reported Outcomes. 2020 Jun 1;4(1):42.

3. Kleijweg JHM, Verbraak MJPM, Van Dijk MK. The clinical utility of the Maslach Burnout Inventory in a clinical population. Psychol Assess. 2013 Jun;25(2):435–41.

4. Schaufeli WB, Bakker AB, Hoogduin K, Schaap C, Kladler A. On the clinical validity of the maslach burnout inventory and the burnout measure. Psychol Health. 2001 Sep;16(5):565–82.

5. Sexton JB, Adair KC. Forty-five good things: a prospective pilot study of the Three Good Things well-being intervention in the USA for healthcare worker emotional exhaustion, depression, work-life balance and happiness. BMJ Open. 2019/03/23 ed. 2019 Mar 20;9(3):e022695.

6. Adair KC, Rodriguez-Homs LG, Masoud S, Mosca PJ, Sexton JB. Gratitude at Work: Prospective Cohort Study of a Web-Based, Single-Exposure Well-Being Intervention for Health Care Workers. J Med Internet Res. 2020 May 14;22(5):e15562.

7. Sexton JB, Schwartz SP, Chadwick WA, Rehder KJ, Bae J, Bokovoy J, et al. The associations between work-life balance behaviours, teamwork climate and safety climate: cross-sectional survey introducing the work-life climate scale, psychometric properties, benchmarking data and future directions. BMJ Qual Saf. 2017 Aug;26(8):632–40.

8. Adair KC, Quow K, Frankel A, Mosca PJ, Profit J, Hadley A, et al. The Improvement Readiness scale of the SCORE survey: a metric to assess capacity for quality improvement in healthcare. BMC Health Services Research [Internet]. 2018 [cited 2019 May 6];18(1). Available from: https://bmchealthservres.biomedcentral.com/articles/10.1186/s12913-018-3743-0

9. Rehder KJ, Adair KC, Hadley A, McKittrick K, Frankel A, Leonard M, et al. Associations Between a New Disruptive Behaviors Scale and Teamwork, Patient Safety, Work-Life Balance, Burnout, and Depression. Jt Comm J Qual Patient Saf. 2019/11/11 ed. 2020 Jan;46(1):18–26.

10. Adair KC, Kennedy LA, Sexton JB. Three Good Tools: Positively reflecting backwards and forwards is associated with robust improvements in well-being across three distinct interventions. The Journal of Positive Psychology. 2020 Jul 9;1–10.

11. Andresen EM, Malmgren JA, Carter WB, Patrick DL. Screening for depression in well older adults: evaluation of a short form of the CES-D (Center for Epidemiologic Studies Depression Scale). Am J Prev Med. 1994 Apr;10(2):77–84.

12. Seligman MEP, Steen TA, Park N, Peterson C. Positive psychology progress: empirical validation of interventions. Am Psychol. 2005 Aug;60(5):410–21.

13. Profit J, Adair KC, Cui X, Mitchell B, Brandon D, Tawfik DS, et al. Randomized controlled trial of the “WISER” intervention to reduce healthcare worker burnout. J Perinatol. 2021 Sep;41(9):2225–34.

14. Rehder K, Adair KC, Sexton JB. The Science of Health Care Worker Burnout: Assessing and Improving Health Care Worker Well-Being. Archives of Pathology & Laboratory Medicine. 2021 Aug 30;145(9):1095–109.

15. Schwartz SP, Adair KC, Bae J, Rehder KJ, Shanafelt TD, Profit J, et al. Work-life balance behaviours cluster in work settings and relate to burnout and safety culture: a cross-sectional survey analysis. BMJ Qual Saf. 2019;28(2):142–50.

16. Tawfik DS, Shanafelt TD, Dyrbye LN, Sinsky CA, West CP, Davis AS, et al. Personal and Professional Factors Associated With Work-Life Integration Among US Physicians. JAMA Network Open. 2021 May 27;4(5):e2111575.

17. Lyubomirsky S, Lepper HS. A Measure of Subjective Happiness: Preliminary Reliability and Construct Validation. Social Indicators Research. 1999 Feb 1;46(2):137–55.

18. Howell RT, Rodzon KS, Kurai M, Sanchez AH. A validation of well-being and happiness surveys for administration via the Internet. Behav Res Methods. 2010 Aug;42(3):775–84.
